# Soft and hard tissue changes after compensatory treatment in skeletal class III malocclusion

**DOI:** 10.1371/journal.pone.0322551

**Published:** 2025-05-07

**Authors:** Lijuan Liu, Yuting Liu, Kai Guo, Haojie Ma, Fanghong Yang

**Affiliations:** 1 Department of Orthodontics, Weifang People’s Hospital, Shandong Second Medical University, Weifang, Shandong Province, China; 2 Department of stomatology, Weifang maternal and Child Health Hospital, Weifang, Shandong Province, China; 3 School of Stomatology, Shandong Second Medical University, Weifang, Shandong Province, China; International Medical University, MALAYSIA

## Abstract

**Background:**

The camouflage treatment of skeletal class III malocclusion can include both premolar extraction and mandibular third molar extraction-based approaches. This study aimed to compare an all four second premolar extraction approach with a mandibular third molar extraction and temporary anchorage devices (TADs)-based approach for compensatory orthodontic treatment in mild to moderate skeletal class III malocclusion.

**Methods:**

31 subjects (mean age = 19.65 ± 3.91 years, male = 12, female = 19) with skeletal class III malocclusion were included in this retrospective, observational study. Lateral cephalograms taken before and after treatment were used to perform measurements for 7 dental indicators, 9 skeletal indicators, and 5 soft tissue indicators. Statistical analyses were performed to compare the cephalometric measurements between groups.

**Results:**

The patients’ profiles were improved after treatment, and molars reached a neutral occlusal relationship. Dental cephalometric measurements showed that mandibular incisor to mandibular plane angle (IMPA) (-7.73 ± 4.72°), lower incisor–Nasion–B point angle (L1-NB angle) (-8.36 ± 4.57°),and the lower incisor–Nasion–B point distance (L1-NB distance) (-2.02 ± 1.42 mm) all significantly reduced (*P* < 0.05) in the premolar extraction group, while the changes were non-significant in the mandibular third molar extraction group, and the between-group differences were significant. The angle between the long axis of upper incisors and that of lower incisors (U1-L1) increased significantly (7.76 ± 8.55°) in the premolar extraction group but decreased significantly in the mandibular third molar extraction group (-4.64 ± 5.96°) (P < 0.05). Skeletal cephalometric measurements showed that Sella–Nasion–B point angle (SNB), decreased (premolar extraction group: -1.43 ± 0.87°, mandibular third molar extraction group: -0.71 ± 0.73°), A point–Nasion–B point angle (ANB) increased (premolar extraction group: 1.01 ± 0.86°, mandibular third molar extraction group: 1.22 ± 0.93°) and Wits increased (premolar extraction group: 1.39 ± 0.93 mm, mandibular third molar extraction group: 1.00 ± 0.60 mm) significantly in both groups, *P* < 0.05; with a significantly larger decrease in SNB in the premolar extraction group (*P* < 0.05). Soft tissue measurement items showed lower lip eversion (LL-E) decreased (premolar extraction group: 1.77 ± 1.61 mm, mandibular third molar extraction group: 0.76 ± 1.14 mm) and Lip Difference increased (premolar extraction group: 2.30 ± 0.58 mm, mandibular third molar extraction group: (2.01 ± 0.52 mm) significantly in both groups (P < 0.05), with between-group differences non-significant. Analysis of co-variance accounting for the pre-treatment values as the covariate, showed significant effects of the treatment group for the parameters IMPA, L1-NB (mm), L1-NB (°), U1-L1(°), SNA, SNB, and Wits value.

**Conclusion:**

For mild and moderate skeletal class III malocclusion patients, both premolar extraction and mandibular third molar extraction with TAD-based approaches showed good clinical outcomes. The premolar extraction group showed greater SNB angle and compensatory lingual inclination of the lower incisors, along with significant reduction in OP-SN angle in the mandibular third molar extraction group, which contributed to the counterclockwise movement of the occlusal plane. Soft tissue changes mainly included improvements in lower lip protrusion with no significant differences noted between the two groups.

## Introduction

Skeletal class Ⅲ malocclusion is prevalent among Asians[1]. It hurts the physical and mental health of patients and significantly hampers their quality of life [2,3]. The available treatment options for skeletal class III malocclusion include growth modification through orthopedic appliances, camouflage treatment using orthodontic dentoalveolar compensation, and orthognathic surgery but the successful treatment of adult skeletal class III malocclusion remains a challenge [1,2,3]. In addition, correcting skeletal class III malocclusion can be inherently difficult, and the diagnosis and treatment process are challenging. Patients with mild to moderate skeletal malocclusion often refuse orthognathic surgery and instead opt for orthodontic camouflage treatment. In patients with minor bone anomalies in the late pubertal growth phase or adulthood, skeletal malformations can often be sufficiently concealed through dentoalveolar compensation, resulting in a more harmonious and coordinated facial appearance, thereby achieving a relatively satisfactory profile. [4,5,6]. During compensatory orthodontic treatment, clinicians often face the decision of whether or not to extract teeth. This decision is influenced by various factors including the interdigitation of teeth, molar distalization, degree of crowding, the curve of Spee, the type of anchorage, the type of vertical bone surface, the type of sagittal bone surface, Bolton index, periodontal condition, facial soft tissue protrusion, the stage of growth, and aesthetic considerations[7]. In current practice, the widespread use of temporary anchorage devices (TADs) has significantly expanded the possibilities for non-extraction treatment, owing to improved biomechanics. As a result, deciding to extract teeth may be difficult in borderline cases. [8]. Presently, there is a lack of extensive data regarding the comparative outcomes of compensatory dentoalveolar camouflage treatment for skeletal class III malocclusion using extraction versus non-extraction approaches combined with TADs-based mandibular dentition distalization techniques [9]. This knowledge gap highlights the need for further research to evaluate the effectiveness and success rates of these treatment modalities. Cephalometric analysis is a fundamental tool for clinical diagnosis and outcome assessment for orthodontic treatment by allowing objective evaluation of the changes in soft and hard tissues.

In the present study, cephalometric data obtained before and after treatment for patients with mild and moderate skeletal class III malocclusion treated with a premolar extraction approach or mandibular third molar extraction with a TAD-based approach, were analyzed to provide a reference for the clinical treatment of such patients.

## Materials & methods

The present study was designed as a retrospective analysis of clinical and cephalometric data from subjects with skeletal class III malocclusion who underwent compensatory treatment using either all second premolar extractions or using mandibular third molar extraction with a TAD-based approach. The study protocol was approved by the Ethical Review Committee of the Scientific Research Project of Weifang Health and Family Planning Commission (WFWSJK-2020–019) and the Weifang Science and technology development plan project (2022YX003). Since the study was retrospective, the need for separate written informed consent from the study participants was waived by the Ethical Review Committee. All the included subjects were of Chinese ethnicity. This study followed the STROBE (STrengthening the Reporting of OBservational studies in Epidemiology) guidelines for observational studies (S2 File).

### Study subjects and clinical data

#### Study subjects.

A total of 31 patients treated from 2016 to 2022 were selected as the study subjects in the Department of Orthodontics, Weifang People’s Hospital. and divided into two groups, 14 patients in the premolar extraction group (mean age = 20.07 ± 3.87 years) and 17 patients in the mandibular third molar extraction group (mean age = 19.29 ± 4.03 years). The demographic features of the sample are presented in Table 1, and no statistically significant difference in age was observed between the two groups.

**Table 1:** Demographic characteristics of study participants.

	Premolar extraction group (n = 14)	Mandibular third molar extraction group (n = 17)	P value	Total (n = 31)
Age ^*^ (mean ± SD years)	20.07 ± 3.87	19.29 ± 4.03	0.59	19.65 ± 3.91
Gender #	Male = 5, Female = 9	Male = 7, Female = 10	0.20	Male = 12, Female = 19

* Independent samples t-test; # Chi-square test.

#### Inclusion criteria.

(1), Patients with all permanent teeth present, who were treated with 0.22” “slot pre-adjusted edgewise appliance for camouflage treatment of skeletal Class III malocclusion, and had completed the course of treatment with complete clinical data available in the hospital record system (2) Patients having anterior crossbite or opposite edge to edge occlusion at the outset; (3) -4° < ANB < 0°; and the crossbite was not due a premature contact and a subsequent mandibular anterior protrusion. (4) The upper and lower dental arches displaying mild to moderate crowding at the outset (Table 2); (5)’Cases in the fifth to sixth stage of cervical spine maturation stage, according to Baccetti’s growth and development of cervical spine analysis method, at the beginning of treatment.

**Table 2:** Comparison of upper and lower arch crowding between premolar extraction group and the mandibular third molar extraction groups.

	Premolar extraction group (mm)	Mandibular third molar extraction group (mm)	*P* ^*^
Upper dental arch crowding	3.11 ± 0.71	3.12 ± 0.57	0.964
Lower dental arch crowding	2.93 ± 0.51	2.88 ± 0.60	0.822

* Independent samples t-test

#### Exclusion criteria.

(1) Patients with a history of any cleft lip and palate syndromes or endocrine disorders. (2) Patients with congenital abnormalities in the number of teeth and malformations. (3) Patients with a history of maxillofacial tumor or trauma. (4) Patients with a history of fixed appliance or orthognathic/orthopedic treatment. (4) Cases where the IMPA and Upper incisor inclination was suggestive of pseudoclass III were excluded.

### Research methods

#### Data acquisition.

Patient selection was retrospective and patients with complete patient records were selected from the hospital record system from July 1st, 2022. The prevalence rate of class III malocclusion was 14.98% among the cases retrieved from the hospital record system. The sample size of this study was computed using the formula: $\;\;\;\;\;\;\text{ N}=\frac{Z_{1-\alpha /2}^{2}\text{P}\left(1-\text{P}\right)}{\delta^{2}}$ to make a calculation. The confidence level α was set at 0.05 (two-sided), where corresponding $Z_{1-\alpha /2}^{2}$ was 1.96, *P* was set at 14.98%, δ was 15%, and the sample size was calculated as 31. As no loss to follow-up was anticipated considering the retrospective design of the study, the sample size of 31 was not adjusted further.

Lateral cephalometric radiographs were taken before and after treatment (the films before treatment were taken within one week before orthodontic treatment, and the films after treatment were taken on the day of debonding). Zhi bei Cloud computer software (produced by Chengdu Boltzmann Zhibei Technology Company) was used to measure and analyze the soft and hard tissue landmarks and distances for cephalometric analysis. All measurements were taken three times by the same orthodontist, using fixed points. The interval between each measurement was two weeks, and the average of the three measurements was calculated. The complete anonymized raw cephalometric data are available in S1 File.

**Fig 1:**
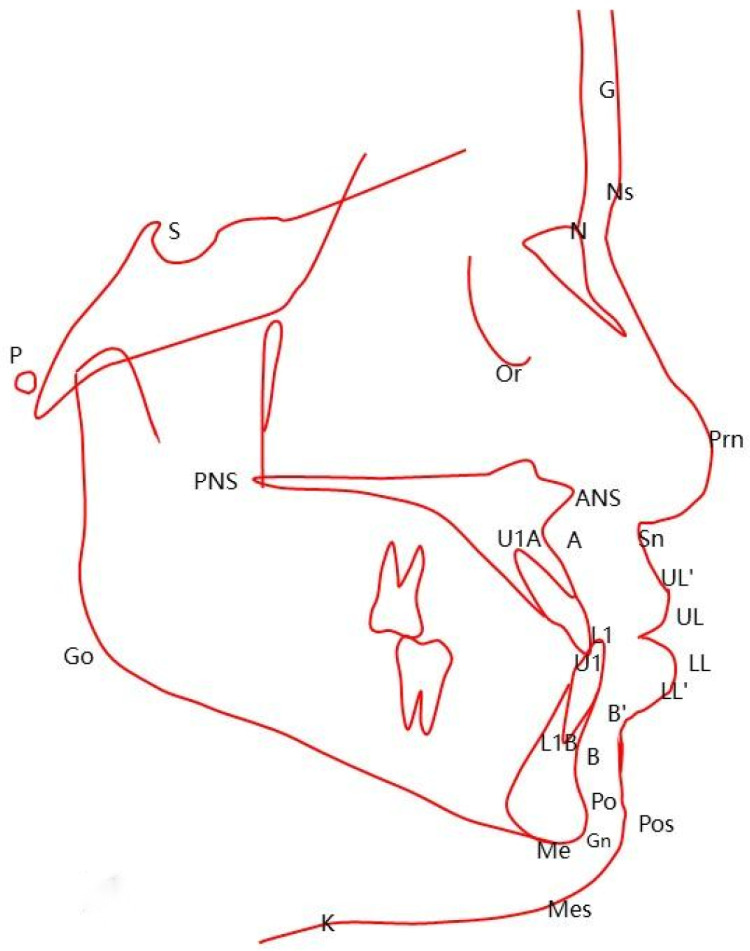
Cephalometric measurement points. S： sella； N： Nasion P： Porion； Or： Orbitale point； Prm: nasal apex; Point A: the deepest point on the curvature of the maxillary alveolar process; UIA: root point of upper central incisor; UI: upper central incisor cutting point; L1: The cutting point of the lower central incisor; Point B: the deepest point in the mandibular symphysis, or the point of the deepest concavity in the lower jaw; LIB: root point of lower central incisor; Pog: Most anterior point on the chin; Gn: mental vertex; Me: submental point; Sn: subnasal point; UL: upper lip bump; UL ‘: upper lip margin point; LL: lower lip bump; LL ‘: lower lip margin point; Pos: soft tissue mental preeminence; Mes: soft tissue submental point; Go: mandibular Angle point.

**Fig 2:**
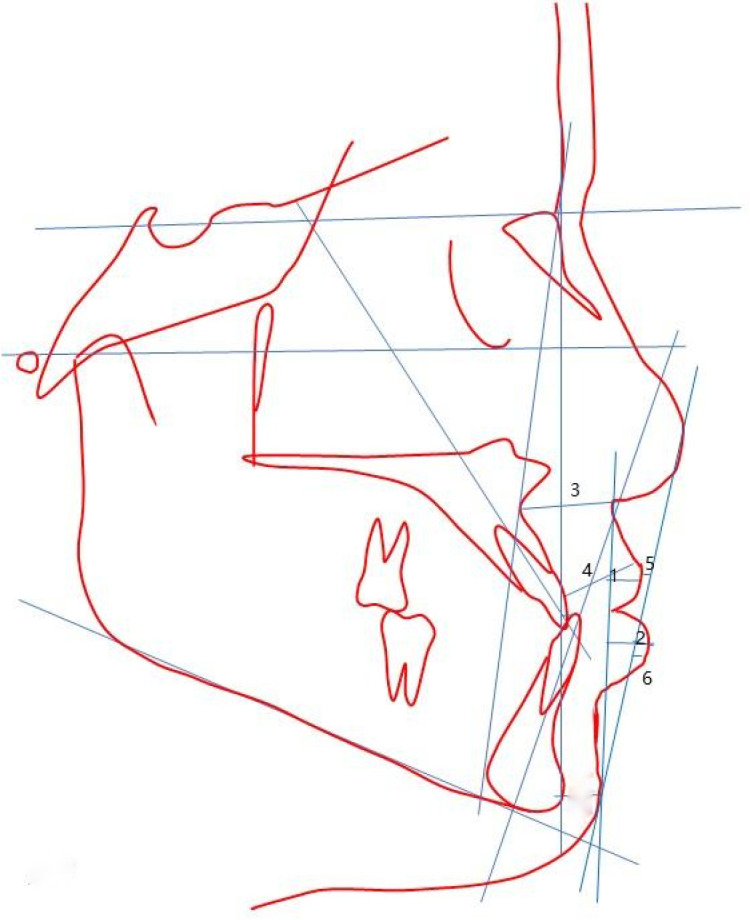
Typical measurement parameters. 1：UL-SnPos(mm)；2：LL-SnPos(mm)；3：Upper lip base thickness；4：Convex upper lip；5：UL-E(mm)； 6: LL-E(mm).

Cephalometric measurement items included seven dental measurement items (U1-SN°, U1-NAmm, U1-NA°, IMPA°, L1-NBmm, L1-NB°, U1-L1°), Nine skeletal measurement items (SNA°, SNB°, ANB°, Witsmm, S-Go/N-Me, MP-SN°, OP-SN°, NA-APo°, SGn-SN°, and Five soft tissue measurement items) UL-Emm, LL-Emm, Lip-Diff mm, Nasolabial angle°, Upper lip tension°) (Figs 1-2). The Lip Difference was UL-SnPos minus LL-SnPos. Upper lip tension was measured as the thickness of the base of the upper lip minus the thickness of the edge of the upper lip.

All 31 patients were bonded with stainless steel, rectangular, self-ligating brackets (SLB) (0.022-in slot, MBT; Shinye™.) to align the upper and lower dental arches. In the mandibular third molar extraction group the mandibular molar movement was achieved using TADs. The preferred site for buccal shelf implant placement was distal to the mandibular second molar. All cases with mandibular third molar extraction received TADs. For premolar extraction cases, a 0.019 × 0.025 stainless steel square wire sliding method was used to close gaps. After the completion of treatment, a retainer was used to maintain the treatment outcome. All patients in the mandibular third molar extraction group had undergone third molar removal prior to treatment.

### Statistical methods

PASS software 2021 (NCSS LLC., Kaysville, U.T., USA) was used for sample-size estimation. The primary endpoint of the study was considered as the difference before and after treatment in the SNB angle. We hypothesized that the difference before and after treatment of the SNB mean differences of the premolar extraction and mandibular third molar extraction groups are 1.1 mm and 0.1 mm, respectively, with a standard deviation of 0.8 based on the preliminary experimental results obtained from the hospital. To yield 80% power to detect an expected improvement based on a two-sided 0.05 significance level test; premolar extraction group: mandibular third molar extraction group = 1:1.21, the sample size was calculated as 11 and 13 for the premolar extraction and mandibular third molar extraction groups, respectively. In anticipation of loss to follow-up (20%), we expected to enroll 31 patients in the present study, with 14 cases in the premolar extraction group and 17 cases in the mandibular third molar extraction group. In addition, analysis of covariance (ANCOVA) was conducted with the change in the cephalometric value (treatment effect) as the dependent variable, and the treatment grouping and pre treatment value as independent predictors. This auxiliary analysis was performed to cater for potential effects of differences in baseline values between the two groups.

## Results

### Cephalometric values compared between the premolar extraction group and the mandibular third molar extraction groups before treatment

As shown in Table 3, there were no statistically significant differences in measurements between the two groups before treatment.

**Table 3:** Comparison of pretreatment cephalometric variables between premolar extraction and mandibular third molar extraction groups.

Variable	Premolar Extraction	Mandibular third molar extraction	*P*#
Dental variables U1-SN(°)	110.6 ± 6.72	108.6 ± 6.23	0.398
U1-NA(mm)	8.15 ± 4.62	6.44 ± 2.75	0.237
U1-NA(°)	28.01 ± 4.97	27.16 ± 4.81	0.631
IMPA(°)	89.33 ± 6.40	86.54 ± 4.02	0.149
L1-NB(mm)	5.19 ± 2.54	4.06 ± 1.66	0.145
L1-NB(°)	24.16 ± 6.92	22.15 ± 4.39	0.335
U1-L1(°)	128.47 ± 10.01	133.66 ± 7.76	0.114
Skeletal variables SNA(°)	82.01 ± 2.23	82.45 ± 2.14	0.581
SNB(°)	83.89 ± 2.32	84.21 ± 2.42	0.707
ANB(°)	-1.81 ± 1.39	-1.74 ± 1.35	0.883
Wits(mm)	-3.65 ± 1.40	-4.20 ± 0.99	0.211
S-Go/N-Me(%)	67.64 ± 4.62	69.13 ± 9.16	0.586
MP-SN(°)	31.48 ± 3.46	31.60 ± 4.42	0.934
OP-SN(°)	13.36 ± 4.11	15.70 ± 3.46	0.095
NA-APO(°)	-4.29 ± 4.61	-4.20 ± 4.31	0.958
Soft Tissue variables Y-axis(°)	59.58 ± 2.67	59.72 ± 2.75	0.883
UL-E(mm)	-2.41 ± 2.69	-2.69 ± 0.98	0.715
LL-E(mm)	1.41 ± 3.04	-0.54 ± 2.32	0.052
Lip-Diff(°)	-1.15 ± 0.47	-0.91 ± 0.38	0.130
Nasolabial angle(°)	91.19 ± 8.58	93.28 ± 10.74	0.559
Upper lip tension(°)	0.51 ± 2.62	0.35 ± 2.55	0.870

#Independent samples t-test

As shown in Table 4, after compensatory orthodontic treatment, IMPA (°) and L1-NB (°), L1-NB (mm) decreased significantly in the premolar extraction group alone, and this change was significantly different between groups. The change in U1-L1 and SNB was significant for both premolar extraction and the mandibular third molar extraction groups, with a significant difference between the two treatment groups. The change in ANB and Wits was also significant for both groups, but this did not vary significantly between the groups. A significant decrease in OP-SN (°) after treatment was seen in the mandibular third molar extraction group. Soft tissue measurement items LL-E(mm) and Lip Difference were statistically significant after treatment in both groups, without any significant inter-group difference.

**Table 4:** Comparison of treatment effects between premolar extraction and mandibular third molar extraction groups. * p < 0.05; ** p < 0.01.

Variable	Premolar Extraction	*P*	Mandibular third molar extraction	*p*+	*P++*
Dental variables U1-SN(°)	-0.44 ± 6.59	0.80	1.79 ± 3.82	0.07	0.27
U1-NA(mm)	-0.91 ± 2.78	0.24	0.58 ± 2.47	0.34	0.12
U1-NA(°)	-0.07 ± 5.44	0.96	1.03 ± 2.66	0.13	0.50
IMPA(°)	-7.73 ± 4.72	<0.01**	-2.19 ± 5.18	0.10	<0.01*
L1-NB(mm)	-2.02 ± 1.42	<0.01**	-0.44 ± 0.88	0.06	<0.01**
L1-NB(°)	-8.36 ± 4.57	<0.01**	-2.48 ± 4.71	0.05	<0.01**
U1-L1(°)	7.76 ± 8.55	0.01*	-4.64 ± 5.96	0.01*	<0.01**
Skeletal variables SNA(°)	-0.34 ± 1.05	0.26	0.28 ± 0.55	0.05	0.06
SNB(°)	-1.43 ± 0.87	<0.01**	-0.71 ± 0.73	<0.01**	0.02*
ANB(°)	1.01 ± 0.86	<0.01*	1.22 ± 0.93	<0.01**	0.51
Wits (mm)	1.39 ± 0.93	<0.01**	1.00 ± 0.60	<0.01**	0.18
S-Go/N-Me(%)	-0.2 ± 0.77	0.35	-0.57 ± 2.11	0.28	0.54
MP-SN(°)	0.68 ± 1.32	0.08	0.43 ± 1.13	0.14	0.58
OP-SN(°)	0.39 ± 3.42	0.68	-1.86 ± 3.33	0.04*	0.08
NA-APO(°)	0.66 ± 1.83	0.20	1.11 ± 4.16	0.29	0.71
Y-axis(°)	-0.13 ± 1.16	0.69	0.51 ± 1.67	0.23	0.24
Soft Tissue variables UL-E(mm)	-0.02 ± 1.65	0.96	0.22 ± 0.47	0.07	0.60
LL-E(mm)	-1.77 ± 1.61	<0.01**	-0.76 ± 1.14	0.01*	0.05
Lip-Diff(°)	2.30 ± 0.58	<0.01**	2.01 ± 0.52	<0.01**	0.16
Nasolabial angle(°)	-0.01 ± 4.13	0.99	-0.57 ± 6.03	0.70	0.77
Upper lip tension(°)	-0.49 ± 2.39	0.45	-0.86 ± 2.19	0.13	0.66

“+”-Paired t-test was used to compare before and after treatment values in each group. “++” The independent sample t-test was used to compare the treatment effects between the premolar extraction and the mandibular third molar extraction group.

### Analysis of Co-variance (ANCOVA) for change in parameter predicted by treatment group with pre-treatment value as a covariate

The results of ANCOVA for change in treatment measures with treatment group and pre-treatment value as predictors showed that, after accounting for the differences in pre-treatment value, treatment group induced significant effects for the outcomes IMPA(°), L1-NB(mm), L1-NB(°), U1-L1(°), SNA(°), SNB(°), and Wits, consistent with the previous results, except for SNA(°) (Table 5).

**Table 5:** Results of Analysis of Co-variance (ANCOVA) for change in parameter predicted by treatment group with pre-treatment value as a covariate. * p < 0.05; ** p < 0.01.

Variable	Pre-treatment Value: β, p-value	Treatment Group: β, p-value
Dental Variables U1-SN(°)	-0.56, p < 0.01**	1.13, p = 0.44
U1-NA(mm)	-0.47, p < 0.01**	0.69, p = 0.35
U1-NA(°)	-0.35, p = 0.03*	0.81, p = 0.57
IMPA(°)	-0.22, p = 0.22	4.92, p = 0.01*
L1-NB(mm)	-0.34, p < 0.01*	1.20, p < 0.01*
L1-NB(°)	-0.38, p < 0.01*	5.11, p < 0.01**
U1-L1(°)	-0.51, p < 0.01*	-9.77, p < 0.01**
Skeletal variables SNA(°)	-0.05, p = 0.50	0.63, p = 0.04*
SNB(°)	0.03, p = 0.63	0.71, p = 0.02*
ANB(°)	-0.12, p = 0.33	0.23, p = 0.49
Wits(mm)	-0.49, p < 0.01**	-0.66, p < 0.01**
S-Go/N-Me(%)	-0.56, p < 0.01*	-0.13, p = 0.76
MP-SN(°)	0.02, p = 0.67	-0.25, p = 0.58
OP-SN(°)	-0.22, p = 0.19	-1.72, p = 0.18
NA-APO(°)	-0.32, p = 0.02*	0.48, p = 0.68
Y-axis(°)	-0.19, p = 0.06	0.66, p = 0.20
Soft Tissue variables UL-E(mm)	-0.39, p < 0.01**	0.14, p = 0.68
LL-E(mm)	-0.28, p < 0.01**	0.46, p = 0.32
Lip-Diff(°)	-0.21, p = 0.03*	-0.10, p = 0.95
Nasolabial angle(°)	-0.43, p = 0.02*	-0.31, p = 0.11
Upper lip tension(°)	-0.76, p < 0.01**	-0.48, p = 0.27

The ‘premolar extraction’ group was considered as the reference group for the predictor ‘Treatment Group’.

### Typical cases

#### Case with premolar extraction.

**Fig 3:**
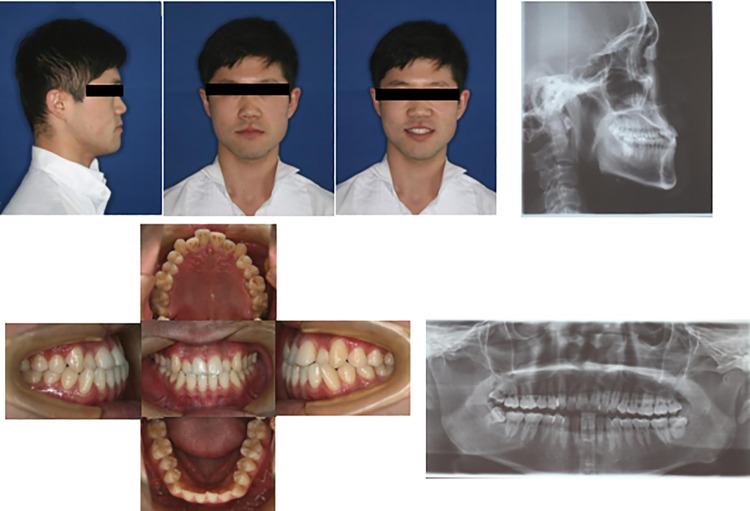
Pretreatment facial, intraoral photographs, and radiographs of the case with premolar tooth extraction. These showed that the anterior mandibular process and bilateral molar relationships were mesial relationships with a crossbite.

**Fig 4:**
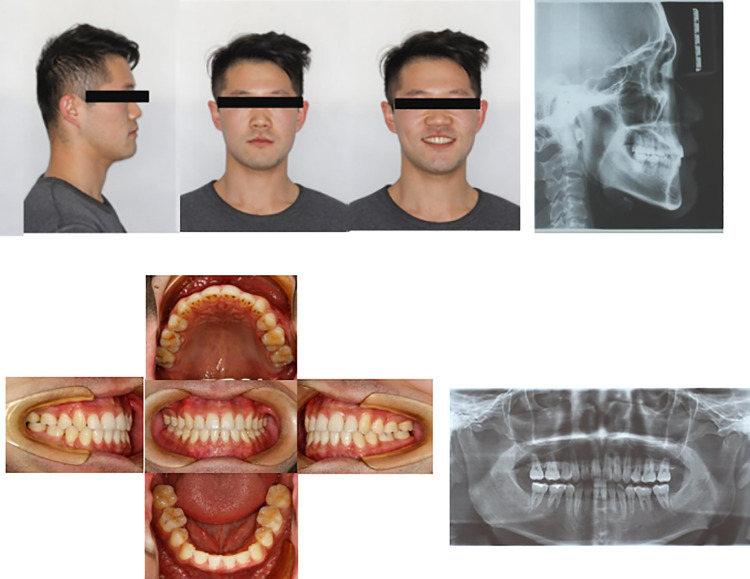
Posttreatment facial, intraoral photographs, and radiographs of the case with premolar tooth extraction. These showed that bilateral molar relationships were neutral, with normal overbite.

**Fig 5:**
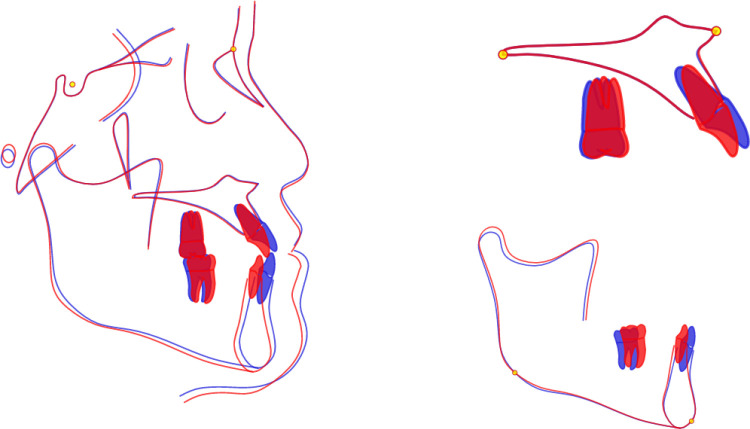
Superimposition of the cephalometric tracings obtained before and after treatment of case with premolar tooth extraction. These showed differences between dental, skeletal, and soft tissue parameters before and after treatment.

The treatment of a 28-year-old male, complaining of irregular teeth is depicted (Figs 3–5). Examination showed slight overcrowding of upper and lower dentition, crossbite occlusion of 11, 12, 22, 32, 41 and 42, shallow overbite of front teeth, and normal relationship of bilateral molars. The overjet and overbite were 0.2 mm and 0.5 mm, respectively. ANB was -2.6°. The lower lip showed greater protrusion relative to the upper lip. The patient did not receive TADs, and all four second premolar tooth extractions were performed. The treatment plan was to remove the bilateral second premolars, and MBT straight wire arch orthodontics was used to align the upper and lower dental arches. A 0.019 × 0.025 stainless steel rectangular wire was used, with intermaxillary traction for space closure, followed by fine adjustment. At completion, bilateral molar relationships were neutral, the anterior teeth showed normal overbite, and the patient’s profile was improved. After appliance removal, a retainer was placed.

#### Case with mandibular third molar extraction.

**Fig 6:**
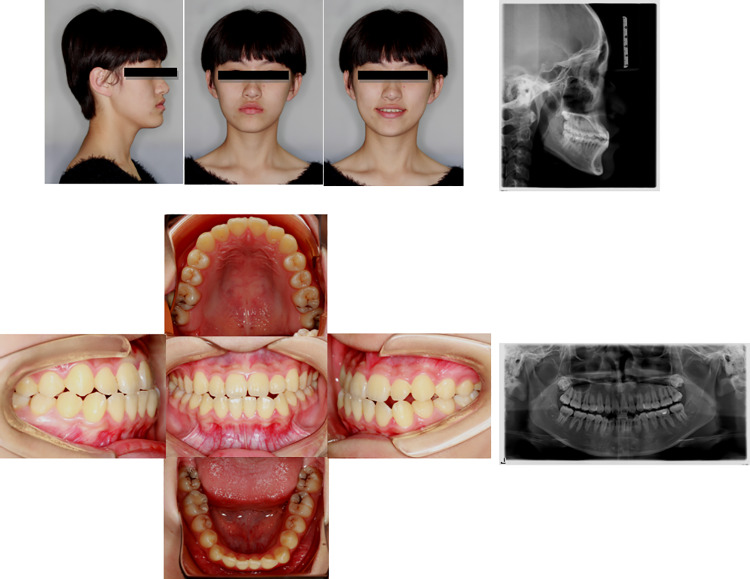
Pretreatment facial, intraoral photographs, and radiographs of the case with mandibular third molar extraction. These showed that the anterior mandibular process and bilateral molar relationships were mesial relationships with crossbite.

**Fig 7:**
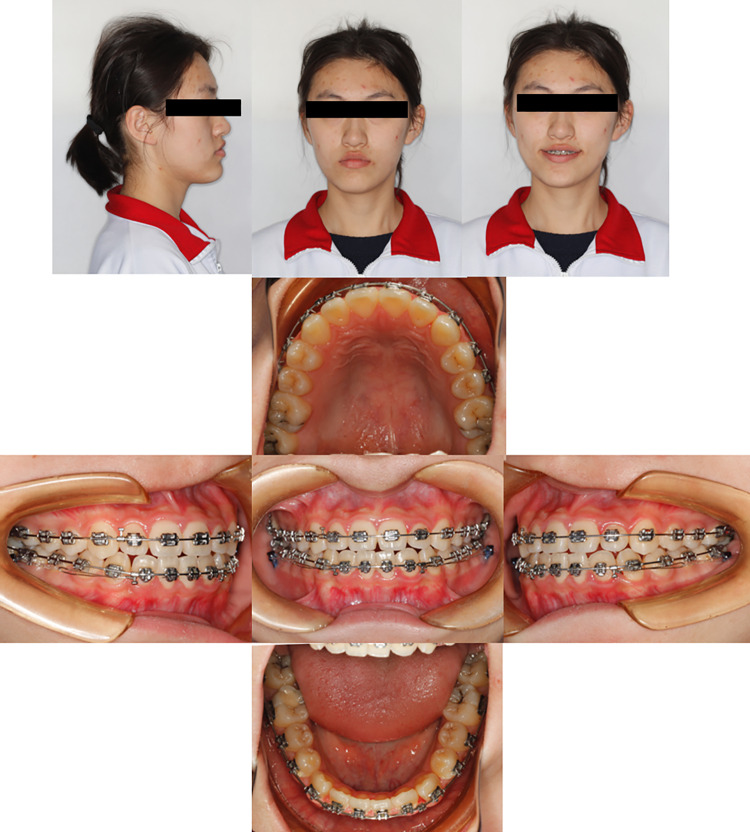
Intraoperative facial and intraoral photographs of the case with mandibular third molar extraction. These show that after one year of treatment, the lower dentition was successfully distalized using TADs. From the intraoral photograph b, it can be seen that the overbite of the lower anterior teeth was improved.

**Fig 8:**
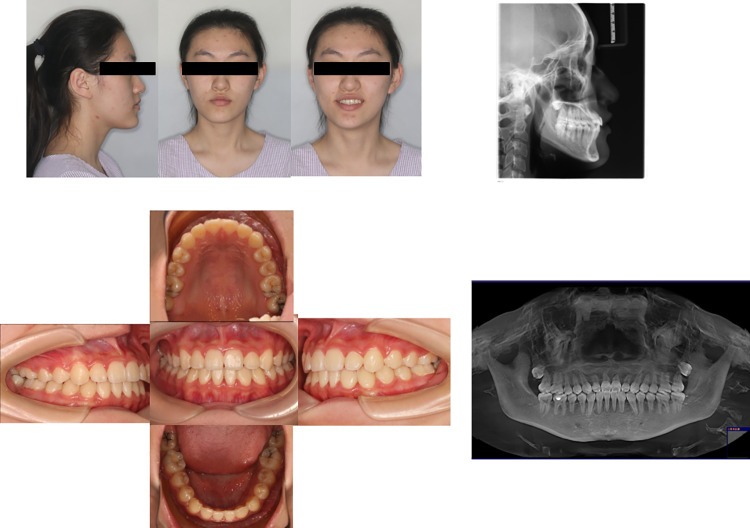
Posttreatment facial, intraoral photographs, and radiographs of the case with mandibular third molar extraction. The bilateral molars were in neutral relationships, the anterior teeth showed normal overbite relationship and the profile was improved.

**Fig 9:**
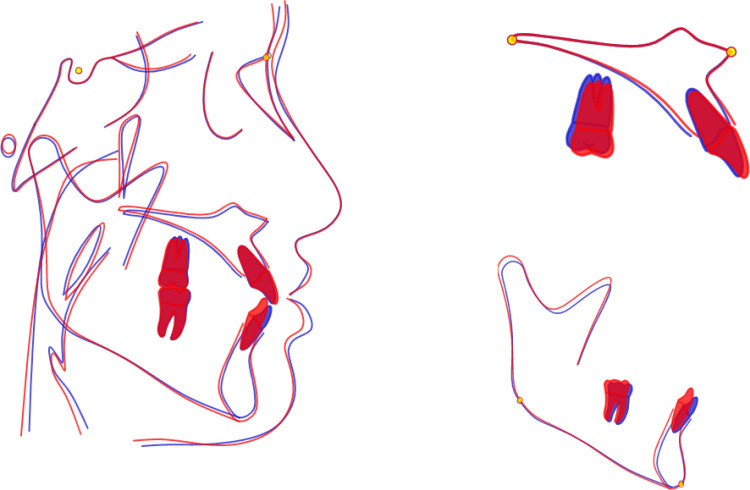
Superimposition of the cephalometric tracings obtained before and after treatment of case with mandibular third molar extraction. These showed differences between dental, skeletal, and soft tissue parameters before and after treatment.

A 16 year old female patient complained of irregular teeth and protrusion of front teeth and compensatory treatment was performed without premolar extraction and mandibular third molar extraction (Figs 6–9). Examination revealed permanent dentition, slight crowding of upper and lower dentition, crossbite occlusion of 11, 12, 21, 22, 23, 31, 32, 33, 41 and 42, with reversed overbite. The overjet and overbite were -0.3 mm and 0.2mm, respectively. ANB was -1.4°. The patient’s mandibular third molars had been removed. The patient showed a greater protrusion of the lower lip relative to the upper lip. She received TADs and chose not to extract premolar teeth for orthodontic correction. The treatment plan included MBT straight wire arch orthodontics to align the upper and lower dental arches, and two TADs (Ormco™, 2.0mm X 10mm) were implanted in the mandible to distalize the lower dentition. A 0.019 × 0.025 stainless steel rectangular wire was combined with intermaxillary traction for molar distalization, followed by fine adjustment. Post-treatment, bilateral molar relationships were found to be neutral, the anterior teeth showed a normal overbite relationship, and the patient’s profile was improved. After the removal of the fixed orthodontic appliance, retainers were placed.

## Discussion

In adult skeletal class III malocclusion, as active jaw growth has ceased, compensatory camouflage orthodontic treatment poses significant challenges. At present, the objective of compensatory orthodontic treatment is to address the discrepancy between the upper and lower jaws by adjusting the position of the teeth, aiming to achieve more harmonious dental esthetics. The present retrospective observational study showed that in non-growing patients with mild to moderate skeletal class III malocclusion who underwent camouflage treatment, both second premolar extraction and mandibular third molar extraction with TADs achieved satisfactory outcomes.

Both groups of patients exhibited a buccal inclination of the upper anterior teeth before treatment and a lingual inclination of the lower anterior teeth with different degrees of dental compensation. To prevent potential negative impacts on periodontal health caused by excessive buccal inclination [10], tooth extraction and correction were considered. For patients with concave skeletal malocclusion, the development of the maxilla may often be inadequate. Improper treatment in these cases can result in a more flattened facial appearance. Therefore, careful planning for extraction treatment is warranted and the second premolar teeth were selected for extraction in this study[11,12,13]. While mandibular first premolar extraction is a more common approach, it may create a larger extraction space. This can pose challenges in achieving proper torque control of the lower incisors, particularly in skeletal class III camouflage cases [14]. When the extraction space is greater than the space required to correct crowding, forward movement of the posterior teeth is warranted, and second premolar extraction may be advantageous for maintaining the facial morphology to avoid a flattened facial appearance [15]. In an earlier report, Zhou et al. [11] showed that U1-SN decreased by 5.6°and and U1-NA decreased by 4.0° after the removal of the second premolar for dental compensation treatment for skeletal class III malocclusion. In this study, U1-SN decreased by 0.44° and U1-NA decreased by 0.07°in the premolar extraction group (Table 4), and ANCOVA showed a significant effect of the pre-treatment value on the magnitude of change. Variations in the buccal inclination of anterior teeth, skeletal surface type, and soft tissue parameters could also account for these differences. U1-SN and U1-NA, representing maxillary incisor inclination relative to the cranial base, were non-significantly increased in the mandibular third molar extraction group. Although TADs were used in the mandibular third molar extraction group, factors such as higher maxillary crowding in some cases, thinner bone in the mandibular symphysis region, and thicker mucosa at the mandibular posterior region may have contributed to the treatment mechanics and outcomes. While the buccal inclination of the maxillary anterior teeth in the mandibular third molar extraction group increased slightly after treatment, the difference was not statistically significant, thus excessive inclination of the maxillary incisors and the consequent compromise in facial esthetics was successfully prevented [16]. It is important to note that the maxillary development was largely normal in the present study sample and the subnasal point was relatively less depressed, which enabled the possibility to achieve treatment results with a moderate buccal tilt of the anterior teeth.

In skeletal class III malocclusion, thin and lingually inclined bone is frequently noted at the buccolingual aspect of lower anterior teeth, and typically, the greater the degree of skeletal discrepancy, the lower the thickness of the alveolar bone on the lingual aspect of lower anterior teeth [17], which imposes limitations on the compensatory tooth movement possible [18]. Therefore, the compensatory movement of lower anterior teeth in skeletal class III cases should be moderate to prevent the risk of iatrogenic fenestration [19]. In both treatment groups, the lower anterior teeth showed a compensatory lingual inclination. The compensatory lingual inclination in the premolar extraction group was slightly greater than that in the mandibular third molar extraction group but the difference was non-significant (Table 3), suggesting a lack of significant bias in case selection. After treatment, both groups maintained a compensatory lingual inclination of lower incisors, and a normal overbite was achieved. Earlier data has shown that the compensatory potential of the lower incisors is a very important factor in the non-surgical treatment of skeletal class III malocclusion [20]. In all cases included in the present study, the retroclination of the lower incisors was significantly increased but the root remained within the cortical bone of the symphysis. After treatment. In skeletal class III patients treated with camouflage orthodontic treatment, the root may not be located in the center of the alveolar bone as a whole [21], so a small range of movement may be safely feasible [21], where tipping movement can be more favorable for establishing the apical fossa relationship between the upper and lower teeth in mild and moderate skeletal deformities [22].

The maxillary position of the patients included in this group was largely normal, and the mandible was overdeveloped. The ANB Angle was about -2.0° before treatment in both groups, which remained negative after treatment, but the Wits value increased significantly, suggesting that the facial profile was improved after the treatment, which may be due to the change in the position of point B due to the movement of the lower incisor teeth and alveolar bone changes in the anterior mandible [23]. TADs have been applied to treat skeletal class III malocclusion by distalizing the lower dentition without premolar tooth extraction [24]. Here, the increase in ANB angle and Wits value were comparable in both groups. The vertical indices of the two groups did not change significantly after treatment, which was consistent with the results of Zhou et al. [11] and Fan et al. [24], suggesting that the improvement of overbite in patients was mostly the result of dental compensation. The counterclockwise rotation of the occlusal plane noted in the mandibular third molar extraction with TADs (non-premolar extraction group) was consistent with past research results [25–29]. Yeon et al. [28] compared mandibular buccal TADs and a titanium plate in the mandibular ramus to visualize teeth to treat skeletal class III malocclusion and found that buccal TADs led to greater counterclockwise rotation of occlusal surface by molar intrusion and less distal mandibular movement. When traction force is parallel to the functional occlusal plane, remote molar movement is more effective [27]. These findings suggest that when using TADs to assist the treatment of skeletal class III malocclusion patients, the vertical skeletal pattern should be considered in deciding the most optimal placement of temporary anchorage devices, to control the direction of traction force. Of note, the positioning of TADs in the present retrospective study was performed based on individual case evaluation and not standardized.

Soft tissue changes are also important in long-term outcomes of orthodontic treatment and a change of profile is an important index to measure the treatment outcome [30,31]. Among Han Chinese, the lip soft tissue morphology of patients with skeletal class III malocclusion tends to show a short and thin upper lip with retraction and pronation and high tension of the upper lip musculature, while the lower lip is typically long and convex with weak muscle tone [32]. In this study, the upper lip tension in both the premolar extraction and the non-premolar extraction group was relatively high before treatment, and a small change was seen after treatment without any significant inter-group difference. In patients with skeletal class III malocclusion, the lower lip is usually relatively protrusive compared to the upper lip, and a concave profile significantly impacts facial aesthetics. Lip difference has been proposed as one of the evaluation indicators for the treatment efficacy of skeletal malocclusion [33]. Chen et al. [34] showed that the degree of convexity of the upper and lower lips was a sensitive measure of the efficacy of skeletal class III malocclusion treatment. Here, after treatment, the lip difference in both the premolar extraction and the non-premolar extraction groups changed from a negative value to a positive value, reflecting significant improvement in the sagittal mismatch of the upper and lower lips, indicating that the lip relationship was closer to harmony. Of note, the nasolabial angle of the premolar extraction group was largely unchanged, which further indicated that the upper lip retained good fullness despite dental retraction. The nasolabial angle of the mandibular third molar extraction group decreased, but the difference after treatment was not statistically significant, indicating that while the lip inclination did increase slightly, an acceptable lip shape was maintained. After treatment, the difference between the lower lip and upper lip position was reduced. The lower lip was retracted effectively, and the facial soft-tissue profile improved in both groups [29,35] with no significant between-group difference, indicating comparable facial soft-tissue-related outcomes in both groups. No significant differences in lower lip retraction were noted between the two groups. Previous research found that as compared with non-premolar extraction therapy, the premolar extraction group resulted in greater profile changes, including those in the protrusion of the upper lip [36]. In the present study, the treatment-associated soft tissue changes were attributed mainly to the change in the lower lip position, while the upper lip position did not change significantly. The results of ANCOVA indicated that the change in lip difference was significantly affected by the baseline value and not by the treatment group. At the same time, it is notable that these results were measured shortly after removal of the appliance, and treatment stability was not assessed, so long-term differences may exist between premolar extraction and mandibular third molar extraction groups, which remain to be investigated.

In summary, the present data indicated that the discrepancy in bone and soft tissue in the two groups of non-growing mild to moderate skeletal class III malocclusion patients were camouflaged to varying degrees, and a normal overbite and occlusion was established [37], leading to relatively good facial aesthetics. This main finding was consistent with earlier data [38] showing no significant difference between the facial contour changes between groups treated with and without extraction of premolars. Notably, skeletal discrepancies persist after non-surgical treatment of class III malocclusion. In case the growth and development are incomplete in patients selected for orthodontic compensation, the treatment outcome may be compromised by a relatively high recurrence rate. In addition, the risk of recurrence in skeletal class III patients treated with tooth extraction is reportedly lower [39] but treatment stability was not assessed in the present study design. Post-treatment stability is an important issue in patients with distalization of molars and adequate post-extraction space can provide more dental compensation than non-extraction treatment [40]. Possibly, successful orthodontic compensation treatment of borderline class III malocclusion depends chiefly on the implementation of a comprehensive orthodontic treatment plan that accurately analyzes individual characteristics such as bone and soft tissue, as opposed to the mere choice of premolar extraction or non-extraction and TADs. The main implication of these findings is that both premolar extraction and mandibular third molar extraction with TADs based distalization can be considered for skeletal class III cases which may be borderline candidates for orthognathic surgery based on case-specific analysis and patient’s desires.

However, these findings must be considered in light of the limitations. The major limitations of the present study include a small sample size, restricted inclusion criteria and a retrospective study design, which preclude the extrapolation of these findings. Of note, the sample size analyzed in this study was for an observational study design to assess treatment effects of compensatory treatment in skeletal class III malocclusion. The data from this observational retrospective study for mean differences in change from baseline scores for the two treatment groups can inform the design for adequately powered randomized controlled trials to validate these preliminary findings. Other criteria that must be analyzed in future studies include the provision of orthopedic therapy in adolescence, incisor or molar extractions and varying treatment mechanics for vertical and horizontal growth patterns. Another consideration is the possible confounding by pseudo skeletal class III malocclusion cases [41], which was addressed in the present study by careful clinical and radiographic diagnosis and ANB angle assessment before inclusion. The patients included in this study had a mean age of less than 20 years. As some male patients may show continued growth after this age, the cervical spine maturation stage was standardized for case-selection purposes, which is a reliable indicator of jaw growth [42]. However, the treatment outcomes must be observed over a longer period. In particular, the lack of randomization imposes biases in terms of case allocation subject to clinical assessment and patients’ preferences. Large-sampled studies with prospective designs, randomization, and assessment of 3-D imaging data are essential to further dissect this subject. Well-controlled trials describing comparative outcomes of camouflage treatment versus more invasive treatment modalities in this domain, and specific clinical criteria for case selection are lacking. Furthermore, patient-reported outcomes (PROMS) are an important outcome of premolar extraction versus mandibular third molar extraction and TAD’s based compensatory treatment that needs to be analyzed. Overall, such data can aid the development of objective case-selection criteria for different approaches in the camouflage management of skeletal class III malocclusion.

## Conclusions

This observational, retrospective study showed that for patients with mild and moderate skeletal class III malocclusion patients, both premolar extraction and mandibular third molar extraction with TADs-based camouflage treatments could achieve satisfactory clinical outcomes. In the premolar extraction group, there was a greater compensatory lingual inclination of the lower incisors. A larger change was observed in SNB in the premolar extraction group along with significant reduction in OP-SN angle in the mandibular third molar extraction group due to counterclockwise movement of the occlusal plane. Regarding soft tissue changes, the main differences were seen in the recovery of lower lip protrusion and lip position, while no significant difference in soft tissue changes was observed between the two groups. Larger-sampled, randomized controlled prospective clinical trials are warranted to validate these findings.

## Supporting information

S1 File Anonymized raw cephalometric data. This Excel file contains the complete dataset of all pre-treatment and post-treatment cephalometric measurements collected from the 31 subjects (14 in premolar extraction group and 17 in mandibular third molar extraction group), including all dental, skeletal, and soft tissue parameters analyzed in this study.
(XLSX)

S2 File STROBE checklist. This document contains the completed STrengthening the Reporting of OBservational studies in Epidemiology (STROBE) checklist that was followed in conducting this retrospective observational study.
(DOCX)

## References

[1] Soh J, Sandham A, Chan YH. Occlusal status in Asian male adults: prevalence and ethnic variation. Angle Orthod. 2005;75(5):814–820. doi: 10.1043/0003-3219(2005)75[814:OSIAMA]2.0.CO;2 16279828

[2] Palomares NB, Celeste RK, Miguel JA. Impact of orthosurgical treatment phases on oral health-related quality of life. Am J Orthod Dentofacial Orthopedics. 2016;149(2):171-181. 10.1016/j.ajodo.2015.07.032 26827973

[3] Javed O, Bernabe E. Oral impacts on quality of life in adult patients with Class I, II and III malocclusion. Oral Health Prev Dent. 2016;14(1):27-32 26106654 10.3290/j.ohpd.a34377

[4] Sevillano MGC, Diaz GJF, de Menezes LM. Management of the vertical dimension in the camouflage treatment of an adult skeletal Class III malocclusion. Case Reports in Dentistry. 2020;2020:8854588. doi: 10.1155/2020/8854588 32850154 PMC7441420

[5] Alhaija E.S., Al-Khateeb S.N. Skeletal, dental and soft tissue changes in Class III patients treated with fixed appliances and lower premolar extractions. Australian Orthodontic J. 2011;27(1):40–45. doi: 10.2478/aoj-2011-0008 21696113

[6] Watanabe J-H, Fitarelli F, de Freitas D-S, Cançado R-H, de Oliveira R-C-G, de Oliveira R-C-G, et al. Comparison of the facial profile attractiveness in Class III borderline patients after surgical or compensatory orthodontic treatment. J Clin Exp Dent. 2020;12(4):e348–e353. doi: 10.4317/jced.56750 32382384 PMC7195682

[7] Zhao Z., Li X. Thinking of treatment for orthodontic borderline cases. Chinese J Pract Stomatol. 2012;6(2):65-70.

[8] Krishnaswamy NR. Contemporary solutions for managing Class III malocclusion. J Indian Orthod Soc. 2015;49:19-26.

[9] Almalki R.A., Alhazmi A., Devanna R. Evaluation of skeletal class III treatment with mini-screw-a systematic review. Saudi J Oral Dent Res. 2022;7(10):261—269.

[10] Nance HN. The removal of second premolar in orthodontic treatment. Am J Orthod. 1949;35(9):685—695. 18139241 10.1016/0002-9416(49)90125-6

[11] Zhou YQ, Yang ZP, Teng F. Class Ⅲ malocclusion treated by second premolar extraction. Chinese J Orthodont. 2017;24(2):115—120.

[12] Chen D.P., Xu L., Bo Z.Y. Effects of second premolar extraction on profile prominence in borderline cases. Chinese J Orthodont. 2007;14(4):157—161.

[13] De Launay L, Gebeile-Chauty S. The smile: a challenge in the treatment of Class III. Orthodontie Francaise. 2018;89(1):81–91. doi: 10.1051/orthodfr/2018002 29676256

[14] Ngoc VTN, Phuong NTT, Anh NV. Skeletal class III malocclusion with lateral open bite and facial asymmetry treated with asymmetric lower molar extraction and lingual appliance: a case report. Int J Environ Res Public Health. 2021;18(10):5381. doi: 10.3390/ijerph18105381 34070132 PMC8158363

[15] Yang L, Bai D. Application of second premolars extractions in orthodontic treatment. Int J Stomatol, 2007, 34(03): 226-228.

[16] Kim Y, Park JU, Kook YA. Alveolar bone loss around incisors in surgical skeletal Class III patients. Angle Orthodontist. 2009;79(4):676—682. 19537864 10.2319/070308-341.1

[17] Ko Y-I, Baek S-H, Mah J. Determinants of successful chincup therapy in skeletal Class III malocclusion. Am J Orthod Dentofacial Orthop. 2004;126(1):33–44. doi: 10.1016/j.ajodo.2002.12.003 15224056

[18] Yao C-C, Chang Z-C, Lai H-H, Hsu L-F, Hwang H-M, Chen Y-J. Architectural changes in alveolar bone for dental decompensation before surgery in Class III patients with differing facial divergence: a CBCT study. Sci Rep. 2020;10(1):1-1. doi: 10.1038/s41598-020-71126-3 32873841 PMC7463229

[19] Oh S.H., Nahm K.Y., Kim S.H., Nelson G. Alveolar bone thickness and fenestration of incisors in untreated Korean patients with skeletal Class III malocclusion: a retrospective 3-dimensional cone-beam computed tomography study. Imaging Sci Dent. 2020;50(1):1–14. doi: 10.5624/isd.2020.50.1.9 32206615 PMC7078404

[20] Tseng Y-C, Pan C-Y, Chou S-T, Liao C-Y, Lai S-T, Chen C-M, et al. Treatment of adult Class III malocclusions with orthodontic therapy or orthognathic surgery: receiver operating characteristic analysis. Am J Orthod Dentofacial Orthop. 2011;139(5):485-493. doi: 10.1016/j.ajodo.2010.12.014 21536190

[21] Andrews L.F., Andrews W.A. The six elements of orofacial harmony. Andrews J. 2000;1:13-22.

[22] Zhang X, Gao J, Sun W. Evaluation of alveolar bone morphology of incisors with different sagittal skeletal facial types by cone beam computed tomography: a retrospective study. Heliyon. 2023;9(4):e15369. doi: 10.1016/j.heliyon.2023.e15369 37113777 PMC10126934

[23] Sharma JN. Skeletal and soft tissue point A and B changes following orthodontic treatment of Nepalese Class I bimaxillary protrusive patients. Angle Orthod. 2010;80(1):91–96. doi: 10.2319/010409-6.1 19852646 PMC8978738

[24] Fan SQ, Zhou YH. Evaluation of the correction of the skeletal Class III malocclusion by distalization of the whole mandible dentition with micro-implant anchorage. J Peking Univ. 2010;49(3):91–96. 28628160

[25] Cui Y, Zhao ZH, Zhao Q. Treatment effects of distal movement of lower arch with miniscrews in the retromolar area compared with miniscrews in the posterior area of the maxillary. J Craniofacial Surg. 2013;24(6):1974—1979. 10.1097/SCS.0b013e3182a248ae 24220385

[26] Oh Y-H, Park H-S, Kwon T-G. Treatment effects of microimplant-aided sliding mechanics on distal retraction of posterior teeth. Am J Orthod Dentofacial Orthop. 2009;139(4):470–481. doi: 10.1016/j.ajodo.2009.05.037 21457858

[27] Kook Y-A, Park JH, Bayome M. Distalization of the mandibular dentition with a ramal plate for skeletal Class III malocclusion correction. 2016;152(2):364–377. 27476370 10.1016/j.ajodo.2016.03.019 27476370

[28] Yeon BM, Lee N-K, Park JH. Comparison of treatment effects after total mandibular arch distalization with miniscrews vs ramal plates in patients with Class III malocclusion. Am J Orthod Dentofacial Orthop. 2021;161(4):529–536. doi: 10.1016/j.ajodo.2020.09.040 34953658

[29] Park H-S, Yoon D-Y, Park C-S, Jeoung S-H. Treatment effects and anchorage potential of sliding mechanics with titanium screws compared with the Tweed-Merrifield technique. Am J Orthod Dentofacial Orthop. 2008;133(4):593–600. doi: 10.1016/j.ajodo.2006.02.041 18405824

[30] Abu Alhaija ES, Al-Khateeb SN. Skeletal, dental and soft tissue changes in Class III patients treated with fixed appliances and lower premolar extractions. Aust Orthod J. 2011;27(1):40–45. doi: 10.2478/aoj-2011-0008 21696113

[31] Hershey HG, Smith LH. Soft-tissue profile change associated with surgical correction of the prognathic mandible. Am J Orthod. 1974;65:483–502. doi: 10.1016/0002-9416(74)90031-1 4524314

[32] Yang YF, Liang Y, Wang YL. An retrospective study of soft tissue changes of lip and chin morphology in skeletal Class III adult patients with orthognathic surgery. Chinese J Orthod. 2015;3(5):132-136.

[33] Lin JX, Gu Y. Preliminary investigation of nonsurgical treatment of severe skeletal Class III malocclusion in the permanent dentition. Angle Orthodontist. 2003; 73(4):401-410. 12940561 10.1043/0003-3219(2003)073<0401:PIONTO>2.0.CO;2

[34] Chen H, Su H, Han B. The application of lip difference in the evaluation of the soft tissue change among different skeletal malocclusions with non-surgical treatment. Chinese Journal of Orthodontics. 2017;24(3):2-9.

[35] Zimmer B, Gaida S, Dathe H. Compensation of skeletal Class III malocclusion by isolated extraction of mandibular teeth: Part 2: skeletal, dentoalveolar and soft tissue parameters in comparison with nonextraction Class III therapies. J Orofac Orthop. 2016;77(2):119–128. doi: 10.1007/s00056-016-0016-6 26935962

[36] Xu TM, Yang MZ, Huang W. Comparison of extraction versus non-extraction orthodontic treatment results--a preliminary study. West China J Stomatol. 2003;21(3):205-207. 12898764

[37] Yang FH, Han XY, Li WJ. Primary evaluation of the correlation between discrepancy index and orthodontic case treatment effectiveness evaluation index in orthodontic borderline cases. Chinese J Orthodontics. 2020;27(3):158-161.

[38] Iared W, Koga da Silva EM, Iared W, Rufino Macedo C. Esthetic perception of changes in facial profile resulting from orthodontic treatment with extraction of premolars. Journal of the American Dental Association. 2017;148(1):9–16. doi: 10.1016/j.adaj.2016.09.004 27771001

[39] Blagitz MN, Almeida G., Normando D. Factors associated with the stability of compensatory orthodontic treatment of Class III malocclusion in the permanent dentition. Am J Orthod Dentofacial Orthop. 2020;158(5):e63–72. doi: 10.1016/j.ajodo.2020.06.030 33131569

[40] Chung K-R, Kim S-H, Choo H, Kook Y-A, Cope JB. Distalization of the mandibular dentition with mini-implants to correct a Class III malocclusion with a midline deviation. Am J Orthod Dentofacial Orthop. 2010;137(1):135–146. doi: 10.1016/j.ajodo.2007.06.023 20122441

[41] Al-Hummayani FM. Pseudo Class III malocclusion. Saudi Med J. 2016;37(4):450–456. doi: 10.15537/smj.2016.4.13685 27052290 PMC4852025

[42] Manabe A, Ishida T, Kanda E, Ono T. Evaluation of maxillary and mandibular growth patterns with cephalometric analysis based on cervical vertebral maturation: a Japanese cross-sectional study. PLoS One. 2022;17(4):e0265272. doi: 10.1371/journal.pone.0265272 35385488 PMC8985984

